# Tuning high-*Q* nonlinear dynamics in a disordered quantum magnet

**DOI:** 10.1038/s41467-019-11985-1

**Published:** 2019-09-05

**Authors:** D. M. Silevitch, C. Tang, G. Aeppli, T. F. Rosenbaum

**Affiliations:** 1 Division of Physics, Mathematics, and Astronomy, California Institute of Technology, Pasadena, California 91125 USA; 2 Department of Physics, ETH Zurich, Zurich, CH-8093 Switzerland; 3 Department de Physique, EPF Lausanne, Lausanne, CH-1015 Switzerland; 4 Paul Scherrer Institut, Villigen, PSI CH-5232 Switzerland

**Keywords:** Magnetic properties and materials, Phase transitions and critical phenomena

## Abstract

Quantum states cohere and interfere. Atoms arranged imperfectly in a solid rarely display these properties. Here we demonstrate an exception in a disordered quantum magnet that divides itself into nearly isolated subsystems. We probe these coherent spin clusters by driving the system nonlinearly and measuring the resulting hole in the linear spectral response. The Fano shape of the hole encodes the incoherent lifetime as well as coherent mixing of the localized excitations. For the Ising magnet LiHo_0.045_Y_0.955_F_4_, the quality factor *Q* for spectral holes can be as high as 100,000. We tune the dynamics by sweeping the Fano mixing parameter *q* through zero via the ac pump amplitude as well as a dc transverse field. The zero crossing of *q* is associated with a dissipationless response at the drive frequency. Identifying localized two-level systems in a dense and disordered magnet advances the search for qubit platforms emerging from strongly interacting, many-body systems.

## Abstract (web summary)

The Ising magnet LiHo_0.045_Y_0.955_F_4_ allows experimental probes of disordered quantum dynamics. Silevitch et al. show that strongly-driven localized spin clusters form effectively decoupled two-level systems whose interactions and coherence can be tuned by external ac and dc magnetic fields.

## Introduction

Localization in quantum systems remains both fundamental to science as well as to technology. It is a venerable subject, starting with the work of Anderson^1^—whose name is associated with disorder-induced localization—and Mott^2^, whose Mott localization transition is due to repulsion between electrons. Since these pioneering studies, similar concepts have been extended beyond electrons to a wide range of interacting quantum systems. The combined problem of many-body localization (MBL)^3–10^ persists to this day and has inspired recent numerical simulations^11,12^ as well as experiments on cold atoms^13–15^. There is also practical relevance for systems of quantum devices; a notable example is the D-Wave processor^16,17^, which attempts to implement adiabatic quantum computation, but may be limited as a matter of principle by localization effects.

To control these long-lived and independent states, it is necessary to know how they interact with each other and with the outside world. Minimizing the interactions between coherent localized states and the continuum of states in the broader environment is an important goal for realizing an effective quantum computer^18–21^. However, these environmental couplings are by definition weak compared with the transitions among the states contributing to the spectrum for a particular localizing environment, making it difficult to study them directly. Recently, it has been posited that many weak couplings of this sort can be probed by pumping the system into a nonlinear response regime^22^, saturating the discrete transition associated with the coherent state, and resulting in spectral holes. In the present work, we use the Fano lineshapes of the spectral holes to characterize the coupling between the localized subsystems and both external and internal fields. Our work differs from experiments in quantum optics^23^ in that we are examining emergent degrees of freedom in a (magnetic) many-body system rather than weakly coupled (to each other) single-particle states in atoms and semiconductor quantum dots. At the same time, it represents a major advance over our own previous activity on pump-induced Fano resonances in the same magnetic material^24,25^ in that we uncover a remarkably simple phenomenology, including the discovery of a zero crossing for the Fano asymmetry parameter *q*, as a function of nonlinear drive amplitude and quantum mixing via a transverse field.

Asymmetric absorption lineshapes in atomic gases were first addressed by Ugo Fano more than 50 years ago^26^. They arise from interference between discrete transitions in the atoms and ionization into the continuum. Now known as Fano resonances, this formalism has found wide applicability in systems ranging from photo-ionized gases^27^ to high-*T*_c_ superconductors^28^ and photonic crystals^29^ to quantum wells^30^. Their extension to spin liquids provides a means to characterize coherent spin clusters labeled in the time/frequency domain but distributed randomly in space.

The remainder of the paper is structured as follows. We begin by presenting an overview of the experimental system, the dilute Ising magnet LiHo_*x*_Y_1 − *x*_F_4_, focusing on the linear and nonlinear susceptibilities at small *x*. We then describe a pump-probe technique to study these clusters, using the detailed lineshape of the observed Fano resonances to track the evolution of the clusters as a function of external variables such as temperature and applied magnetic field. Most notably, we find that the interaction between the clusters and the incoherent bath of free spins can be tuned continuously to a point where the direct absorption of energy, and hence the dissipation, of the clusters goes to zero. We then discuss the implications of tuning the system into a dissipationless regime coincident with zero crossings of the Fano asymmetry parameter *q* and how these results offer a potential avenue for accessing many-body-localization physics and the possible follow-up experiments that could explore such connections.

## Results

### Review of the experimental system LiHo_*x*_Y_1 − *x*_F_4_

Spin clusters functioning as quantum two-level systems form in the dilute dipolar-coupled magnet LiHo_*x*_Y_1 − *x*_F_4_ under the appropriate thermodynamic conditions^25,31^. The magnetism in this family of rare-earth fluorides has long been studied as a realization of the dipole-coupled *S* = 1/2 Ising model, with the spins carried by the Ho^3+^ ions and the Ising axis lying along the crystallographic *c* axis^32,33^. The non-magnetic Y^3+^ ions randomly occupy the same sites as the magnetic Ho^3+^ ions with probability 1− *x*. The hierarchy of quantum levels accounting for the charge-neutral excitations of individual Ho^3+^ ions (in the dilute limit where *x* ≪ 1) of this wide-gap insulator has been summarized recently by Matmon et al.^34^. Most relevant for the current low-temperature study are the ground-state doublet for the Ising spins with a crystal-field-derived 9.4 K gap to the first excited state and the hyperfine interaction between the electronic (*J* = 8) and nuclear (*I* = 7/2) spins of the Ho^3+^ ions^32^, which yields a nuclear Zeeman ladder consisting of eight states with spacing 0.2 K between consecutive levels. Analytic solutions of the microscopic Hamiltonian^35–38^, combined with measurements of the crystal-field parameters^32,39^, have quantitatively connected the microscopic Hamiltonian to the long-standing effective Hamiltonian for the spin physics. Magnetic fields applied perpendicular to the Ising axis mix the ground-state doublet with the first excited state, inducing a splitting of the doublet, which in the low-field limit scales as ${\mathrm{\Gamma }} \propto {{H}}_{\mathrm{t}}^2$ (in contrast to the Zeeman splitting $\propto {{H}}_1$ of the ground state in a longitudinal field *H*_l_) and leads to an effective transverse-field Ising Hamiltonian^40,41^:

1 $$H = - \mathop {\sum }\limits_{i,j} J_{ij}\sigma _i^z\sigma _j^z - \Gamma \mathop {\sum }\limits_i \sigma _i^x.$$

In the pure (*x* = 1) limit, a classical ferromagnetic transition occurs at the Curie point *T*_C_ = 1.53 K dictated by the magnitude of the (predominantly) dipolar interactions *J*_*ij*_. The quantum fluctuations induced by the transverse field disorder the Ising spins, producing a quantum critical point at Γ = 1.6 K where the Ising ferromagnetism vanishes. This zero-temperature transition is linked by a line of second-order transitions between paramagnetic and ferromagnetic phases to zero field. The principal features of the dynamics are propagating soft magnetic modes^42^.

Although Eq. (1), which takes no account of the nuclear spins, is an excellent starting point for understanding the physics of pure LiHoF_4_, at temperatures below ~0.6 K the electronic and nuclear spins of the Ho^3+^ ions^32^ combine to form composite degrees of freedom with effective spins *I* + *J*, resulting in an upturn in the ferromagnet–paramagnet phase boundary for pure LiHoF_4_^41^. Furthermore, entanglement of the nuclear and electron spins results in an incomplete softening of the principal magnetic excitation mode at the quantum phase transition^39,42^.

Additional physics has been revealed at holmium concentrations between *x* = 1 for the pure ferromagnet and the *x* ≪ 1 dilute ion limit^43^. The combination of disorder, the magnetic dipole interaction, which can be ferromagnetic or antiferromagnetic (depending on the relative orientation of the preferred spin direction and the vector separating two spins), and quantum fluctuations^44–46^ creates a sequence of states as a function of decreasing dipole (Ho) concentration^43^, progressing from mean-field ferromagnet^41^ to random-field ferromagnet^47^, to spin glass^40,48–50^ to spin liquid^31^. The loss of translational symmetry arising from the dilution creates a local loss of rotational symmetry, which generates internal transverse fields. The quantum fluctuations that arise from these internal transverse fields can act to prevent complete freezing in the *T* → 0 limit even without an externally applied transverse field^51^. Here we focus on the Bhatt–Lee spin liquid^52^ (originally proposed for phosphorus-doped silicon near its metal-insulator transition) for *x* ~ 0.05 and weak thermal coupling, characterized by a hierarchy of singlets derived from combinations of doublets (isolated spins) and triplets (ferromagnetically coupled spins). The hierarchy of singlets results in a low-frequency susceptibility which scales as 1/*T*^*α*^ with *α* ≠ 1 rather than following Curie or spin-glass forms. For LiHo_0.045_Y_0.955_F_4_, *α* was experimentally measured to be 0.75, less than the Curie exponent *α* = 1.0 due to the compensation of spins by each other as they form singlets on cooling^51^. The nature of the ground state for LiHo_0.045_Y_0.955_F_4_ has been a matter of debate, with some groups reporting a spin-glass state^49,53^ and other work indicating a spin-liquid state^31,43^. The discrepancy was resolved in ref. ^25^, which showed that by varying the strength of thermal coupling between the crystal and an external heat bath, it was possible to tune between the two limits. This controlled the rate at which lattice phonons and, subsequently, spins via spin-lattice coupling could exchange energy with the external bath. As discussed below, the measurements reported here were all obtained in the weak thermal-coupling limit, which favors the quantum spin liquid.

**Fig. 1:**
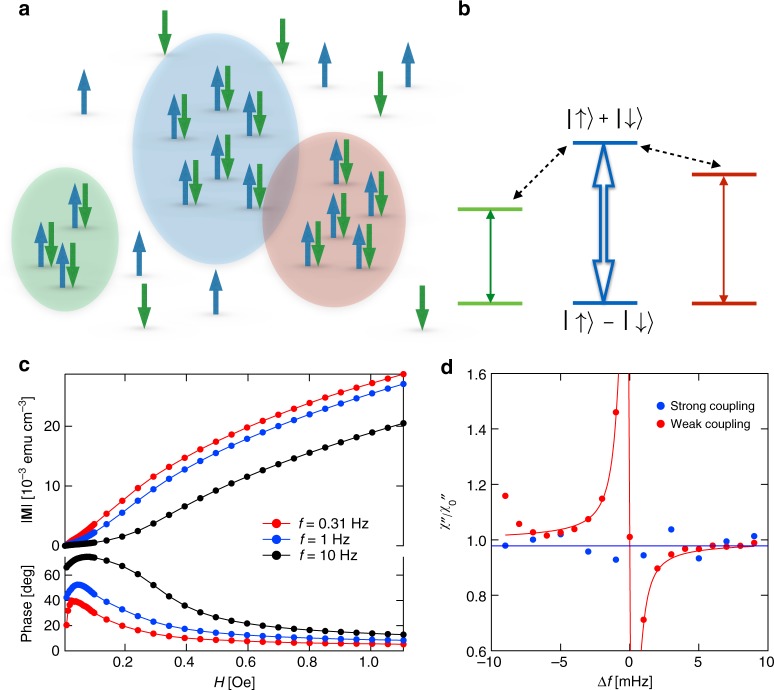
Schematic of spin configuration and transition pathways in pumped LiHo_0.045_Y_0.955_F_4_. **a** Tightly bound spin clusters embedded in a dilute spin bath. Closely separated spins form a tightly bound cluster, which can be magnetized in a nonlinear response regime by a strong ac pump field. Spatially adjacent clusters can interact directly. **b** Schematic of cluster excitation pathways. Clusters of different sizes have different energy gaps, so the choice of pump frequency acts as a size selector. Direct interactions between excited clusters and off-resonance clusters give rise to Fano resonance behavior. **c** Magnetization amplitude (top) and phase angle (bottom) for three different excitation frequencies. **d** Pump-probe measurements (ratios of imaginary part of response χ'' in presence of pump to response χ''_0_ without pump) in two thermodynamic regimes. When LiHo_0.045_Y_0.955_F_4_ is weakly coupled to an external heat reservoir, the low-temperature state is dominated by the quantum cluster response, giving rise to strong Fano resonances. When it is strongly coupled to an external heat reservoir, it exhibits extended glassy behavior including the absence of hole-burning and Fano resonance effects^25^. Both traces were measured at *T* = 100 mK, *f*_pump_ = 20 Hz, and *h*_pump_ = 0.5 Oe

Figure 1a shows schematically how a disordered quantum spin system such as LiHo_*x*_Y_1 − *x*_F_4_ at low concentrations or Si:P^52,54^ breaks into decoupled clusters and isolated spins, focusing on some clusters, whose classical ground states are ferromagnetic but which, because of small transverse fields imposed by other clusters, can be described as two-level systems with low-energy eigenstates that are coherent superpositions of up and down configurations |⇑ > ± |⇓ > = |↑…↑〉 ± |↓↓…↓〉^31^. More elaborate wavefunctions and level schemes^42,55^, taking account of, e.g., electronuclear spin mixing and also classical ground states with antiferromagnetic correlations, will not qualitatively change the physics of hole burning (see Supplementary Note 1) but will be invoked later when we discuss certain details of our data.

### Nonlinear susceptibility of LiHo_0.045_Y_0.955_F_4_

For LiHo_0.045_Y_0.955_F_4_, both the thermal link to the heat bath^25^ and magnetic fields applied transverse to the Ising axis^24^ control the observability of localized excited states. In particular, strong coupling to a thermal bath yields the linear response expected from a spin glass^25,49^, no observable localized excited states, and consequently no evidence for the Fano resonance is seen for strong coupling (Fig. 1d). We therefore concentrate here on weak coupling to the bath.

The first step in the measurements is to determine pump fields *h*_pump_ sufficient to enter the nonlinear regime. Figure 1c shows the magnitude and phase angle of the magnetization induced for a range of frequencies *f*_pump_ limited to a maximum of 10 Hz, to allow measurement to drive fields above 1 Oe without excessive eddy-current heating. The data generally follow an S-shaped curve, whose inflection point moves outward with increasing frequency, in agreement with the model (Supplementary Eq. 1) where the longitudinal field couples the localized singlet ground state of a cluster to an excited state separated by a small gap^51^; the susceptibility at higher frequencies is more sensitive to smaller clusters where the gap is larger, which can only be overcome by a higher drive field *h*_pump_. For our pump-probe experiments, we operate near to and somewhat above the inflection point; very high driving fields are avoided, because they lead to excessive heating. The effective increase in energy of the spin system can be characterized by measuring the shift in the peak frequency of *χ*″(*f*) as a result of nonlinear drive and comparing with linear-response measurements at a range of temperatures. As reported in ref. ^31^, a 5 Hz, 0.2 Oe pump provided energy equivalent to a 40 mK increase in temperature. This can be distinguished from purely thermal effects associated with the drive field by noting the clear change in the dissipative lineshape: the distribution of oscillators visible in *χ*″( *f* ) lacks the tails seen at equilibrium (in the absence of pumping). Although the system consists of an ensemble of spin clusters with a broad range of sizes (previous magnetization measurements found clusters ~250 spins at a 5 Hz resonant frequency), choosing a particular pump frequency selects for a set of clusters that are similarly sized and hence sorted by resonant frequency. These clusters are largely independent of each other and hence pumping at two well-separated frequencies simultaneously results in two distinct holes in the overall spectrum^31^.

### Pump-probe spectroscopy

**Fig. 2:**
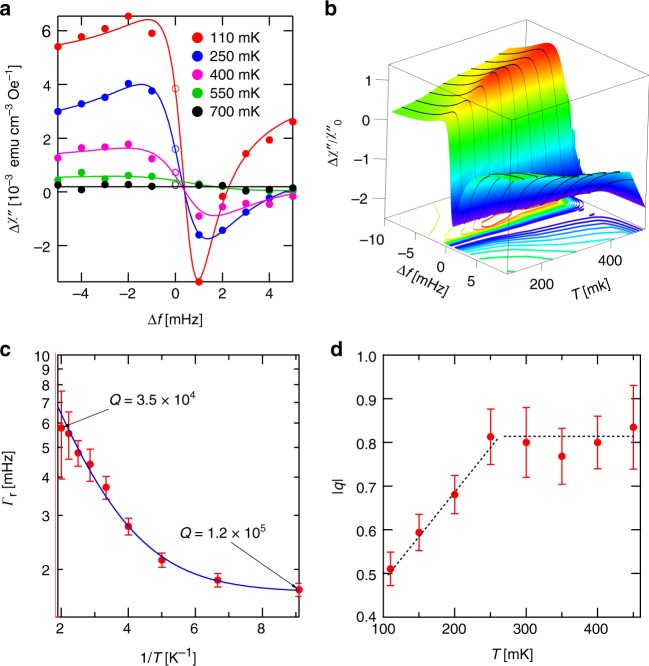
Linear absorption spectra of LiHo_0.045_Y_0.955_F_4_ as a function of temperature. **a** Measured absorption in the presence of a 0.3 Oe pump field at *f*_pump_ = 202 Hz and zero transverse field; probe field has 20 mOe amplitude. Curves are fits to Fano resonance forms, Eq. (2) in the text. The points at *f* = *f*_pump_ (open symbols) are omitted from the fits (see text for details). **b** Absorption normalized with respect to the response at Δ*f* = 30 mHz as a function of frequency and temperature. Color and *z* position both indicate normalized absorption. **c** Linewidth of the resonance, as determined from fitting to the Fano model, vs. temperature *T*. Line is a fit to an intrinsic linewidth of 1.7 mHz plus exponential thermal broadening with *Δ* = 740 mK . **d** Fano parameter *q* vs. temperature, showing the suppression of coupling to the bath at the lowest *T*. Lines are guides to the eye. All error bars are SDs

We turn now to pump-probe measurements, examples of which are shown in Fig. 2a. In these measurements, the system is driven with a strong ac pump field at frequency *f*_0_ and probed with a weak field at a range of frequencies Δ*f* = *f*_probe_ *−* *f*_0_ around *f*_0_ (see Methods for details). The characteristic asymmetric shape of a Fano resonance is immediately obvious, providing a direct indication of a weak coupling between the coherent spin cluster and the continuum of surrounding spin states (Fig. 1b, c). By comparing the integrated area of the resonant response with the area of the entire linear-response spectrum, we estimate that the fraction of spins bound in clusters resonant at the chosen drive frequency is of order 2 × 10^−6^ of the entire sample. As the temperature is increased, the amplitude of the resonant response drops and the resonance appears to broaden, with the response suppressed to 8% of its original amplitude at *T* = 500 mK and to below the noise floor of the measurement at 700 mK. Given that the overall linear susceptibility of LiHo_0.045_Y_0.955_F_4_ has a strong temperature dependence, the thermal evolution of the resonant response can be seen more clearly by normalizing it to the linear response at each temperature (determined by measuring *χ*″(Δ*f* = 30 mHz)). We show in Fig. 2b spectra obtained at a series of temperatures, normalized, and then combined into a surface plot where color and height now represent the absorption for a given Δ*f* and *T*. The broadening of the resonance with increasing temperature emerges clearly in this visualization and we examine it quantitatively in Fig. 2c by looking at the evolution of the linewidth in the fits to the Fano form,

2 $${\mathrm{\chi }}\prime\prime ({{\Delta f}}) = {{A}}\frac{{\left( {\frac{{{{q\Gamma }}}}{2} + {{\Delta f}}} \right)^2}}{{{{\Delta f}}^2 + \frac{{{\mathrm{\Gamma }}^2}}{2}}},$$

where Γ is the linewidth of the resonance and *q*, known as the Fano parameter, characterizes the interference between the different transition pathways. The mHz scale low-temperature limit of the linewidth suggests that the coupling between the clusters and the background spin bath is weak, and hence that the system can be considered in the framework of a two-level system in weak contact with the environment rather than a continuous relaxation process. Quantum states with splittings substantially smaller than nominal bath temperatures are very common in solids and liquids, and indeed form the basis for various resonance (e.g., nuclear magnetic resonance (NMR)) spectroscopies, many of which rely on non-equilibrium quantum-state preparation. Reduced bath coupling during cool-down increases the *T*_1_ and *T*_2_ times associated with such quantum states, and so makes a description of the magnetic response of the system as due to a set of independent multilevel quantum systems more appropriate than a picture based on classical, thermal diffusion.

When a multilevel quantum description applies, for a fixed bath coupling (which extracts heat) and ac driving field (which inserts heat), an equilibrium with a set of fixed state occupancies will characterize the system, and to first order that equilibrium can be described by a fixed effective temperature. On the other hand, the non-equilibrium dynamics are dominated by small multilevel systems describable in terms of some generalized Bloch equation, exactly as is the case, e.g., for NMR performed even at room temperature, and are therefore quantum mechanical. The linewidth increases exponentially with *T*, consistent with a thermally activated process with a gap *Δ* = 740 mK, which is an energy well below the 9.4 K first excited crystal-field state energy but of the same order as nearest-neighbor spin couplings as well as the energy difference^34,41,56^ (~750 mK) between electronuclear states with nuclear moments of 7/2 and 1/2. This suggests that hole burning is favored when Ho nuclear spins are not relaxing relative to electron spins, but does not exclude composite electronuclear wavefunctions involving several Ho ions. Indeed, the fact that several ions need to be involved can be deduced from comparison of the crossover fields (all below 1 Oe) in our ac drive data (Fig. 1c) with the step locations at multiples of 200 Oe in the magnetometry of Giraud et al.^55^. The greatly increased density of level crossings suggested by our data follows naturally from the dipolar interactions between multiple Ho ions. Further indications that the interactions are important come from measurements where changing the thermal boundary conditions vary the state of the system^25^. For strong coupling to an external bath (the mixing chamber of the cryostat), collective spin glass behavior obtains and the nonlinear response also changes qualitatively with no trend towards saturation. In other words, the resonances are not due to single-spin behavior but instead are consistent with a picture consisting of clusters of many spins behaving as a large effective two-level system protected from others in a wavefunction such as that |⇑> ± |⇓> = |↑↑…↑〉 ± |↓↓…↓〉 (discussed above) at low temperatures because of a relatively large single spin-flip energy, associated with either the hyperfine or electronic dipole interactions. In addition to the thermal broadening of the resonance, the lineshape asymmetry *q*(*T*) is also *T*-dependent, varying in approximately linear fashion for *T* < 0.25 K and plateauing above that point (Fig. 2d). A possible microscopic origin for this behavior is that as the temperature grows, thermally activated spin flips will occur within clusters containing antiferromagnetically coupled spins. Such spin flips will result in increased dipolar moments for the clusters and thus an increased coupling to remnant dipole moments of the other clusters forming the underlying bath, an effect seen in the temperature-dependent linewidth shown in Fig. 2c. Provided that a single constant *J*_AFM_ characterizes the underlying antiferromagnetic coupling, we would find that for *k*_B_*T* ≫ *J*_AFM_ the probabilities that two spins are either ferromagnetically or antiferromagnetically correlated become equal and we would see a plateau in the coupling to other clusters as well as the value *q*. Such plateaus also can be found if there is a discrete series of antiferromagnetic couplings *J*_AFM,*i*_ and the conditions *J*_AFM,*i*_ ≪ *k*_B_*T* ≪ *J*_AFM,*i* + 1_ are met. The discrete nature of the distribution of dipolar couplings for the LiHo_*x*_Y_1 − *x*_F_4_ lattice leads to the possibility that these conditions obtain and therefore the data in Fig. 2d, which extend only as far as hole burning can be seen (and *q* can be measured), could be a manifestation of such a plateau.

It should be noted that the free-induction relaxation time of ~10–30 s observed previously^31^ is substantially shorter than the ~500–1000 s of the inverse linewidth of the hole uncovered in the driven pump-probe measurements. This follows because the free-induction decay was measured for relaxation after the strong ac drive field was turned off, whereas the linewidth here is measured in the far more weakly driven linear regime. More formally, the rotating wave approximation^57^ does not apply for the combination of strong (nonlinear) drive fields and low frequency employed for our experiments. In particular, the Rabi frequency *f*_Rabi_ associated with the 0.5 Oe drive field *h*_pump_ for the electronic (Ising-like) spin of a single Ho^3+^ ion is *g*_∥_*μ*_B_*h*_pump_ ~ 10 MHz ≫ *f*_pump_, which is precisely opposite to the requirement that *f*_Rabi_ ≪ *f*_pump_ for the rotating wave approximation to hold.

### Tuning of nonlinear cluster response

**Fig. 3:**
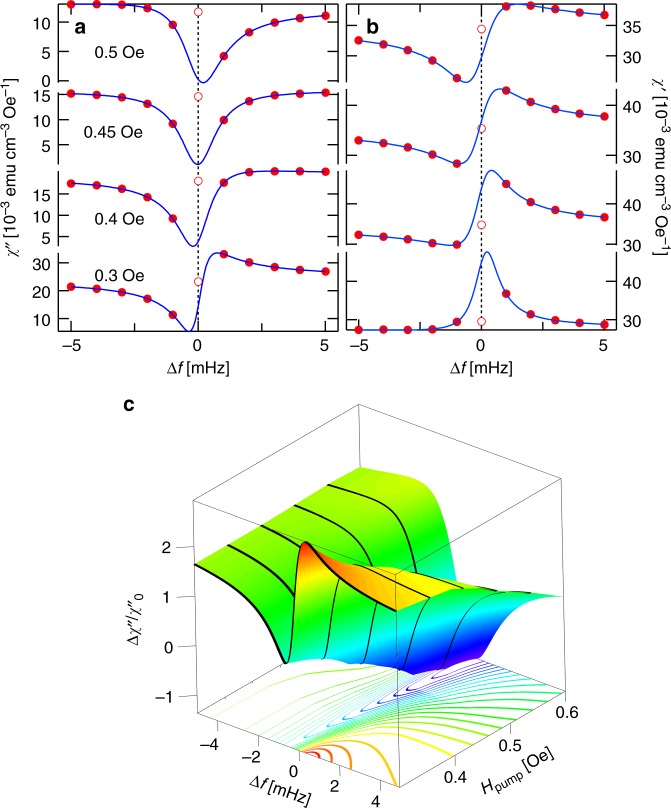
Linear absorption as a function of pump amplitude at *T* = 0.11 K for a 202 Hz pump. **a**, **b** Measured imaginary and real susceptibilities (points) and fits to a Fano resonance form as a function of pump amplitude at zero transverse field. Increasing the pump amplitude tunes the resonant behavior, at the cost of increased decoherence. **c** Normalized absorption as a function of frequency and pump amplitude. Color and *z* position both indicate normalized absorption

**Fig. 4:**
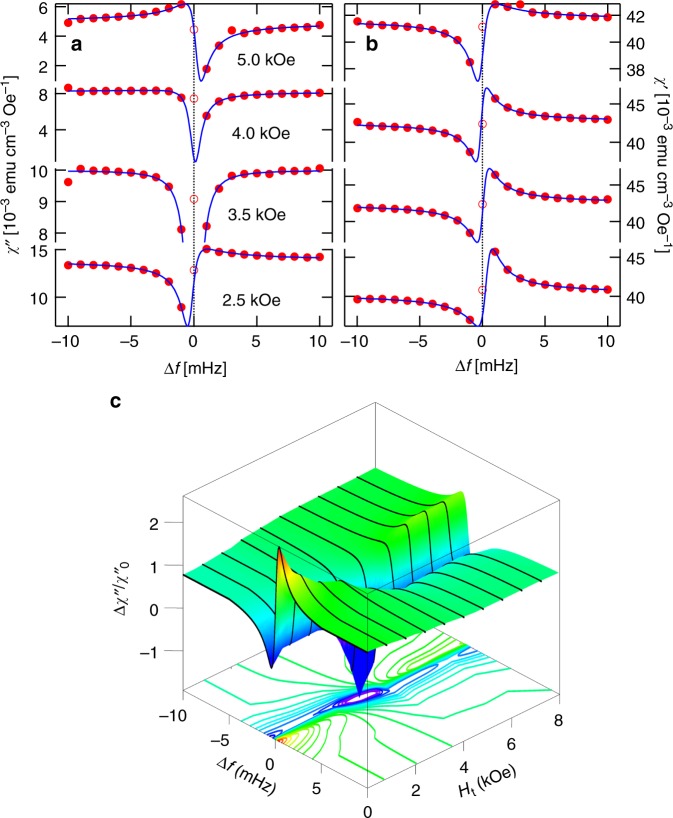
Linear absorption as a function of transverse field at *T* = 0.11 K for a 202 Hz pump. **a**, **b** Measured imaginary and real susceptibilities (points) and fits to a Fano resonance as a function of transverse field for a fixed 0.3 Oe pump. Transverse-field-induced quantum tunneling tunes the resonant behavior without a corresponding increase in decoherence. **c** Normalized absorption as a function of frequency and transverse field. Color and *z* position both indicate normalized absorption

**Fig. 5:**
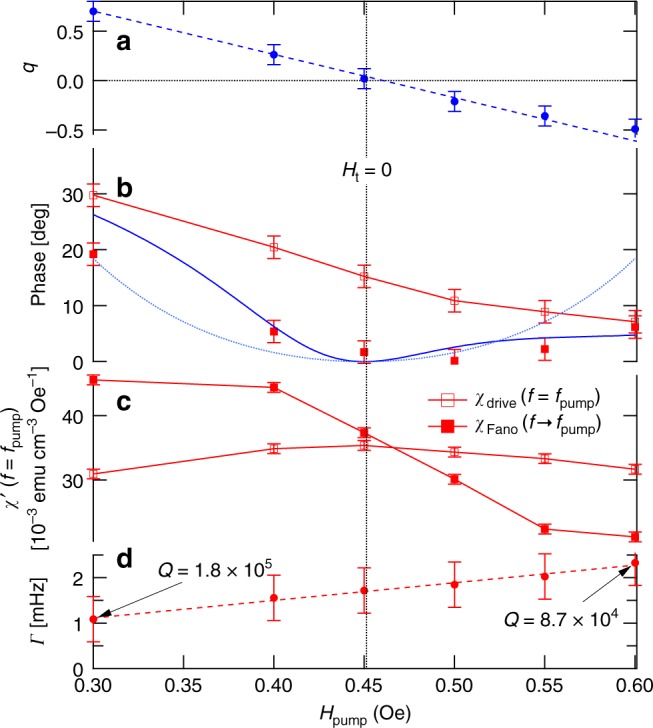
Evolution of resonant behavior vs. pump amplitude at *T* = 0.11 K and *H*_t_ = 0 for a 202 Hz pump. **a** Fano parameter *q* vs. drive amplitude showing a continuous evolution including a smooth crossing through zero. Dashed line is a guide to the eye. **b** Evolution of the phase of the complex susceptibility at *f* = *f*_pump_ for the nonlinear (open symbols) and linear (filled symbols) responses. The zero crossings of *q* are associated with a local minimum in the dissipation and a corresponding minimum in the phase shift of the linear probe response as the probe frequency approaches the pump frequency. $\phi \left( q \right) = \tan ^{ - 1}\frac{{q^2}}{{1 - q^2}}$ (blue dotted curve) follows from Eq. 2 in the text, whereas the solid blue curve (see Eq. 3 and associated discussion in text) provides a better description of the experimental data. **c** Real susceptibility *χ*’ measured directly at *f* = *f*_pump_ (open symbols) and determined by extrapolating the fitted Fano resonance to *f* = *f*_pump_ (filled symbols), showing the contrast in behavior between the nonlinear and linear responses, respectively. **d** Fano linewidth Γ vs. ac drive *h*_pump_. Increasing the drive amplitude broadens the linewidth and hence reduces the oscillator *Q*. All error bars are SDs

**Fig. 6:**
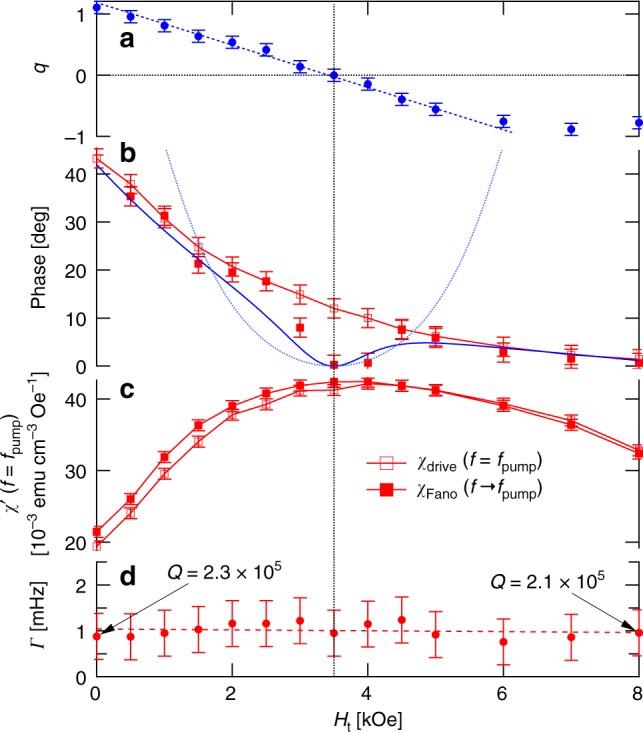
Evolution of resonant behavior vs. transverse field at *T* = 0.11 K for a 202 Hz/0.3 Oe pump. **a** Fano parameter *q* vs. transverse field showing a continuous evolution including a smooth crossing through zero. Dashed line is a guide to the eye. **b** Evolution of the phase of the complex susceptibility at *f* = *f*_pump_ for the nonlinear (open symbols) and linear (filled symbols) responses. As with the *h*_pump_ dependence shown in Fig. 5, the zero crossing of *q* is associated with a vanishing of the dissipation in the linear response with the same functional form, demonstrating universal behavior from two disparate tuning parameters. Blue curves follow from Eqs. 2 and 3 in text as for Fig. 5b. **c** Real susceptibility *χ*’ measured directly at *f* = *f*_pump_ (open symbols) and determined by extrapolating the fitted Fano resonance to *f* = *f*_pump_ (filled symbols), showing a small but apparent distinction in the evolution of the nonlinear and linear responses. **d** Fano linewidth Γ vs. transverse field. In contrast to the behavior as a function of *h*_pump_, increasing *H*_t_ does not change the linewidth. All error bars are SDs

We now examine the effects of various tuning parameters on the cluster response, showing the measured susceptibility spectra in Figs. 3 and 4, and derived quantities in Figs. 5 and 6. We explore in Figs. 3 and 5 the effects of changing the amplitude of the pumping field. Most important is the change in sign of the Fano *q*: for the largest drive field (0.5 Oe), the low- and high-frequency responses are enhanced and suppressed, respectively, opposite to what we see for the lower drive field. The zero crossing of *q* occurs at a critical *h*_pump_ = 0.45 Oe (Fig. 5a). The data point at the pump frequency at which pump and probe-derived signals cannot be distinguished are ignored for the Fano fits, because they represent the response of the highly excited (pumped) clusters and not the perturbatively mixed clusters with other resonant frequencies.

Figure 5b, c reveal clear distinctions between *χ*_drive_, the total signal at *f*_pump_ and *χ*_Fano_, the linear Fano contribution calculated from evaluation at *f* = *f*_pump_ of the fitted Fano form to data at *f* ≠ *f*_pump_. First, $\chi _{{\mathrm{drive}}}^\prime$ goes through a maximum at the zero crossing of *q* (Fig. 5c), whereas $\chi _{{\mathrm{Fano}}}^\prime$ undergoes a decrease that looks like a rounded step. Second, when we plot the phases $\phi = \tan ^{ - 1}\chi \prime\prime /\chi \prime$ (Fig. 5b), we find that although both *χ*_drive_ and *χ*_Fano_ have phase shifts that are smaller at high *H*_drive_, the latter actually has a zero near the zero of *q*. In other words, for small linear perturbations, the Fano response is dissipationless in the limit *f* → *f*_pump_. This result follows from Eq. 2, which gives $\phi = \phi \left( q \right) = \tan ^{ - 1}\frac{{q^2}}{{1 - q^2}}$, a functional form that we superpose over the experimental results in Fig. 5b. The absence of dissipation in the Fano response that describes the linear continuum at *q* = 0 means that hole burning is actually complete at the drive frequency: there is no continuum contribution to *χ*″, which remains unaffected by the drive in the limit *f* → *f*_pump_. Significantly, the absence of dissipation coincident with the *q* zero crossing indicates that the clusters cannot be excited between their ground and first excited states by the external drive. Although *ϕ*(*q*) gives a rough account of the experimental phase angle as *q* moves away from zero, the data ultimately deviate from *ϕ*(*q*), which implies that some oscillators even with frequencies close to *f*_pump_ are not contributing to the Fano profile.

Varying the ac pump amplitude accesses different mixtures of the states of the localized clusters. The additional power applied to the drive solenoid also results in eddy-current-induced heating of the copper susceptometer mount and hence some degree of conductive heating of the sample despite the low-thermal-conductivity Hysol epoxy spacers holding the sample inside the susceptometer. This, as well as dissipation within the sample itself, gives rise to a higher effective temperature, with a concomitant loss of coherence. The decoherence of the resonant excitation is reflected by a measurable increase in the linewidth *Γ*_r_, whose temperature-dependent evolution can be traced readily in Fig. 2c. Over the range of pump amplitudes shown in Fig. 3, the resonance linewidth increases from 1.1 to 1.8 mHz (Fig. 5d), equivalent to ~50 mK of direct thermal heating. Even while heating is clearly present, the line widths remain negligible on the scale of the drive frequency, allowing the coherent superpositions of the cluster and continuum oscillations. Their relative signs change at a critical longitudinal pump field of 0.45 Oe, thus yielding the zero crossing of *q*, one of the main results of our experiment.

We now take advantage of one of the key features of the Li(Ho,Y)F_4_ family, the ability to tune the microscopic Hamiltonian by applying a magnetic field transverse to the Ising axis, thereby quantum-mechanically mixing the single ion and cluster eigenstates^35,40^ via different matrix elements than does the ac longitudinal field. Nonetheless, application of a transverse field induces a crossover (Fig. 4) at a well-defined field of *H*_t_ = 3.5 kOe^24^, similar to that seen earlier as a function of pump amplitude (Fig. 3). We plot in Fig. 6a the transverse field dependence of the Fano parameter *q*. This parameter changes linearly with *H*_t_ over most of the experimental range, showing that the external transverse field not only changes the energies of different states but also tunes their interactions with the broader spin bath environment. In particular, at the *H*_t_ = *H*_c_ = 3.5 kOe crossover field, *q* vanishes. At the same time, as also seen when we varied *h*_pump_ to obtain a zero crossing of *q*, there is a quadratic zero in the phase for *χ*_Fano_(*f*_pump_) (Fig. 6b) and a maximum in $\chi _{{\mathrm{drive}}}^\prime$ (Fig. 6c), both of which coincide with the zero of *q* and can be roughly described by the function *ϕ*(*q*). The simultaneous vanishing of *q* and the phase (and hence the dissipation) opens the possibility for the static transverse field to be used to decouple the localized two-level systems from the external ac field. In contrast to what we saw for the *h*_pump_ scan with *H*_t_ = 0, $\chi _{{\mathrm{drive}}}^\prime$ and $\chi _{{\mathrm{Fano}}}^\prime$ are nearly indistinguishable at *f*_pump_. Another contrast, anticipated from the previous paragraph and visible in the comparison of Figs. 5d and 6d is that the linewidth is, to within error, *H*_t_ independent. The essentially constant behavior of the linewidth is a strong indication that the evolution due to the transverse-field-induced quantum fluctuations is fundamentally different from the purely classical behavior seen as a function of increasing temperature.

## Discussion

It is important to consider the origin of the zero crossings of the Fano *q*—a key result of our experiments—in terms of the ingredients of *q*, which are matrix elements linking the ground and excited states of the resonantly driven spin clusters to each other as well as to the bath formed by other clusters. The measurements characterize the spectral holes inserted by a nonlinear drive field into the continuum of magnetic excitations in a dense set of interacting dipoles. The large but finite lifetime of the excitations is due to a slight mixing between these (almost perfectly) localized excitations and the continuum formed by their ensemble (see Supplementary Note 2). Considering this mixing allows for the proposal of a phenomenological form (solid blue line) for the phase angles plotted in Figs. 5b and 6b:

3 $$\phi \prime \left( q \right) = {\mathrm{tan}}^{ - 1}\left( {\frac{{q^2}}{{q^2 + c^2 + \left( {h_{{\mathrm{pump}}}/d} \right)^2}} \cdot \frac{{\chi _{{\mathrm{drive}}}^{\prime\prime} (f = f_{{\mathrm{pump}}})}}{{\chi _{{\mathrm{drive}}}^\prime (f = f_{{\mathrm{pump}}})}}} \right)$$

(obtained as Eq. 6 in Supplementary Note 2) where *c* = 0.12 represents the intrinsic contributions of the off-diagonal matrix element and *d* = 1.4 Oe incorporates the thermal effects associated with changes in pump amplitude. We note that Eq. (3) explicitly incorporates the experimental observation that the vanishing of the dissipation (and hence of *φ*) coincides with the zero crossing of *q*.

The field scale *H*_c_ can be connected to the microscopic properties of Li(Ho,Y)F_4_ by comparison with an exact diagonalization of the full Hamiltonian for a pair of coupled Ho^3+^ atoms^37^, where there is a crossing of the lowest coupled electronuclear levels for nearest-neighbor spins positioned in the *ab* plane at (100) or (010) relative to each other. The effects of these pairwise level crossings can be seen in the measured linear longitudinal susceptibility *χ*_zz_(*H*_t_)^24,37^. The characteristic dynamics of the pairwise susceptibility shift as a function of classical (temperature) and quantum-mechanical (transverse field) energy scales, moving from 9 kOe at 70 mK to 5 kOe at 150 mK. We expect the same level crossings to impact the dynamics of the larger spin clusters addressed by the pump-probe measurements described here, with the transverse field scales reported here reduced in comparison with those obtained in the pairwise calculation due to additional interactions from further-neighbor spins.

Given that we have not performed similar experiments for compositions *x* other than *x* = 4.5%, we can only speculate as to the possible evolution of the localized cluster behavior as a function of the holmium ion concentration. As *x* is increased to 16.7%, only spin-glass-like behavior has been seen^40,43,58^, suggesting that placing the system in the “antiglass” state, which is a prerequisite for hole burning, would be difficult. By *x* = 20%, a combination of quantum effects and random-field physics for a more nearly ferromagnetic system changes the nonlinear response^59^, and isolated clusters even in the limit of thermal isolation are unlikely and become impossible at the onset of long-range ferromagnetic order at *x* ~ 30%^43^.

Via hole burning, we have demonstrated localization of excitations among interacting magnetic dipoles. For strong coupling to the heat bath, previous work has shown a trend towards spin freezing at low temperatures^25,49^ and the current work (Fig. 1d) shows no spectral hole burning in this strong coupling limit, corresponding to delocalized excitations. Reducing the coupling to the external thermal bath induces a transition to a spin liquid state^25,51^ where the excitations are localized to a very high degree, with *Q* ~ 10^5^ for the spectral hole. The small mixing between excited states of different spin clusters leads to a Fano effect, whose phenomenology for this complex system is remarkably simple, with a vanishing *q* coinciding with an inflection point in the ac magnetization induced by the drive field responsible for the hole burning.

Even if only a subset of the spin clusters in driven LiHo_0.045_Y_0.955_F_4_ display localized excited states, the material with its relatively high density of interacting spins is a promising venue to investigate MBL. As the data have shown, when driven strongly with a “pump” ac magnetic field, select clusters with excitations resonant with the pump frequency can largely decouple from the remaining clusters and can be (almost) fully described by a finite number of local spin operators. The rotating reference frame of the strong pump field suggests the possibility of considering the system in a Floquet picture^60^, where the effective spins of the on-resonant clusters form a network of co-rotating spins, whereas the off-resonant clusters are far more weakly driven and form the continuum of excitations required for the appearance of Fano resonances. By measuring the structure of the resonances, we probe the dynamics of the localized cluster excitations and have shown that the dynamics are tunable both by varying the strength of the strong pump field and by introducing quantum mixing through a static transverse magnetic field. It has been suggested^11,61^ that MBL would be observable in such systems via detailed measurements of the energy spectra of spatially localized operators. In LiHo_*x*_Y_1 − *x*_F_4_, the requisite localized (spin) operators are localized in frequency, which is likely to be equivalent to spatial localization given the broad magnetic response spectrum of this disordered magnet and the very sharp holes burnt by the nonlinear ac drive. Direct measurements of the evolution of the cluster coherence times as temperature approaches zero would be helpful to distinguish between very slow conventional quantum dynamics and actual many-body localization effects.

## Methods

### High-resolution magnetic pump-probe spectroscopy

We cooled a single crystal of LiHo_0.045_Y_0.955_F_4_ in a helium dilution refrigerator and measured its ac magnetic susceptibility for frequencies from 1 to 2000 Hz. The thermal coupling between the crystal and the heat reservoir—the mixing chamber of the dilution refrigerator—was in the weakly coupled regime described by ref. ^25^, thereby maximizing quantum fluctuations. We employed a pump-probe technique^24,25,31^ to access the nonlinear response regime. In this configuration, a two-frequency ac magnetic field is applied along the Ising axis of the crystal. A strong (0.2–0.6 Oe) field *h*_pump_ cos (2*πf*_pump_*t*) excites clusters at the pump frequency *f*_pump_. Simultaneously, a 20 mOe probe field is swept through a range of frequencies to yield a linear response from the crystal. The net response of the crystal is then sensed by an inductive pickup coil. Due to the extremely narrow separation between the pump and probe frequencies (as small as 1 mHz), disentangling the responses required the development of a two-stage lock-in technique, where the combined response is passed into a commercial lock-in amplifier tuned to the probe frequency, and the resulting output is sampled by a computer and passed through a software lock-in detector tuned to Δ*f* = *f*_pump_ − *f*_probe_. It should be noted that the sampling step requires a minimum of 1/Δ*f*  seconds; all of the data reported here were sampled for a minimum of 2/Δ*f*  seconds.

## Supplementary information

Supplementary Information

## Data Availability

The data that support the findings of this study are available from the corresponding author upon reasonable request.

## References

[1] Anderson PW (1958). Absence of diffusion in certain random lattices. Phys. Rev..

[2] Mott NF (1949). The basis of the electron theory of metals, with special reference to the transition metals. Proc. Phys. Soc. A.

[3] Gornyi IV, Mirlin AD, Polyakov DG (2005). Interacting electrons in disordered wires: Anderson localization and low-*T* transport. Phys. Rev. Lett..

[4] Basko DM, Aleiner IL, Altshuler BL (2006). Metal–insulator transition in a weakly interacting many-electron system with localized single-particle states. Ann. Phys..

[5] Oganesyan V, Huse DA (2007). Localization of interacting fermions at high temperature. Phys. Rev. B.

[6] Polkovnikov A, Sengupta K, Silva A, Vengalattore M (2011). *Colloquium*: Nonequilibrium dynamics of closed interacting quantum systems. Rev. Mod. Phys..

[7] Huse DA, Nandkishore R, Oganesyan V, Pal A, Sondhi SL (2013). Localization-protected quantum order. Phys. Rev. B.

[8] Nandkishore R, Huse DA (2015). Many-body localization and thermalization in quantum statistical mechanics. Annu. Rev. Condens. Matter Phys..

[9] Altman E, Vosk R (2015). Universal dynamics and renormalization in many-body-localized systems. Annu. Rev. Condens. Matter Phys..

[10] Alet F, Laflorencie N (2018). Many-body localization: an introduction and selected topics. Comptes Rendus Phys..

[11] Johri S, Nandkishore R, Bhatt RN (2015). Many-body localization in imperfectly isolated quantum systems. Phys. Rev. Lett..

[12] Khemani V, Lim SP, Sheng DN, Huse DA (2017). Critical properties of the many-body localization transition. Phys. Rev. X.

[13] Schreiber M (2015). Observation of many-body localization of interacting fermions in a quasirandom optical lattice. Science.

[14] Choi J-Y (2016). Exploring the many-body localization transition in two dimensions. Science.

[15] Bernien H (2017). Probing many-body dynamics on a 51-atom quantum simulator. Nature.

[16] Johnson MW (2011). Quantum annealing with manufactured spins. Nature.

[17] Harris R (2018). Phase transitions in a programmable quantum spin glass simulator. Science.

[18] Pellizzari T, Gardiner SA, Cirac JI, Zoller P (1995). Decoherence, continuous observation, and quantum computing: a cavity QED model. Phys. Rev. Lett..

[19] Byrd MS, Lidar DA (2002). Comprehensive encoding and decoupling solution to problems of decoherence and design in solid-state quantum computing. Phys. Rev. Lett..

[20] Tyryshkin AM, Lyon SA, Jantsch W, Schäffler F (2005). Spin manipulation of free two-dimensional electrons in Si/SiGe quantum wells. Phys. Rev. Lett..

[21] Ithier G (2005). Decoherence in a superconducting quantum bit circuit. Phys. Rev. B.

[22] Kroner M (2008). The nonlinear Fano effect. Nature.

[23] McCall SL, Hahn EL (1969). Self-induced transparency. Phys. Rev..

[24] Silevitch DM, Gannarelli CMS, Fisher AJ, Aeppli G, Rosenbaum TF (2007). Quantum projection in an Ising spin liquid. Phys. Rev. Lett..

[25] Schmidt MA, Silevitch DM, Aeppli G, Rosenbaum TF (2014). Using thermal boundary conditions to engineer the quantum state of a bulk magnet. Proc. Natl Acad. Sci. USA.

[26] Fano U (1961). Effects of configuration interaction on intensities and phase shifts. Phys. Rev..

[27] Bryant HC (1977). Observation of resonances near 11 eV in the photodetachment cross section of the H^−^ ion. Phys. Rev. Lett..

[28] Thomsen C, Cardona M, Gegenheimer B, Liu R, Simon A (1988). Untwinned single crystals of YBa_2_Cu_3_O_7-δ_: an optical investigation of the a-b anisotropy. Phys. Rev. B.

[29] Poddubny AN, Rybin MV, Limonov MF, Kivshar YS (2012). Fano interference governs wave transport in disordered systems. Nat. Comms..

[30] Faist J, Capasso F, Sirtori C, West K, Pfeiffer L (1997). Controlling the sign of quantum interference by tunnelling from quantum wells. Nature.

[31] Ghosh S, Parthasarathy R, Rosenbaum TF, Aeppli G (2002). Coherent spin oscillations in a disordered magnet. Science.

[32] Hansen P, Johansson T, Nevald R (1975). Magnetic properties of lithium rare-earth fluorides: ferromagnetism in LiErF_4_ and LiHoF_4_ and crystal-field parameters at the rare-earth and Li sites. Phys. Rev. B.

[33] Beauvillain P, Renard JP, Laursen I, Walker PJ (1978). Critical behavior of the magnetic susceptibility of the uniaxial ferromagnet LiHoF_4_. Phys. Rev. B.

[34] Matmon G, Lynch SA, Rosenbaum TF, Fisher AJ, Aeppli G (2016). Optical response from terahertz to visible light of electronuclear transitions in LiYF_4_:Ho^3^. Phys. Rev. B.

[35] Chakraborty PB, Henelius P, Kjonsberg H, Sandvik AW, Girvin SM (2004). Theory of the magnetic phase diagram of LiHoF_4_. Phys. Rev. B.

[36] Schechter M, Stamp PCE (2008). Derivation of the low- T phase diagram of LiHo_x_Y_1−x_F_4_: a dipolar quantum Ising magnet. Phys. Rev. B.

[37] Gannarelli CMS, Silevitch DM, Rosenbaum TF, Aeppli G, Fisher AJ (2012). Contribution of spin pairs to the magnetic response in a dilute dipolar ferromagnet. Phys. Rev. B.

[38] McKenzie RD, Stamp PCE (2018). Thermodynamics of a quantum Ising system coupled to a spin bath. Phys. Rev. B.

[39] Rønnow HM (2007). Magnetic excitations near the quantum phase transition in the Ising ferromagnet LiHoF_4_. Phys. Rev. B.

[40] Wu W, Ellman B, Rosenbaum TF, Aeppli G, Reich DH (1991). From classical to quantum glass. Phys. Rev. Lett..

[41] Bitko D, Rosenbaum TF, Aeppli G (1996). Quantum critical behavior for a model magnet. Phys. Rev. Lett..

[42] Rønnow HM (2005). Quantum phase transition of a magnet in a spin bath. Science.

[43] Reich DH (1990). Dipolar magnets and glasses: neutron-scattering, dynamical, and calorimetric studies of randomly distributed Ising spins. Phys. Rev. B.

[44] Brooke J, Rosenbaum TF, Aeppli G (2001). Tunable quantum tunnelling of magnetic domain walls. Nature.

[45] Schechter M, Stamp PCE (2005). Significance of the hyperfine interactions in the phase diagram of LiHo_x_Y_1−x_F_4_. Phys. Rev. Lett..

[46] Silevitch DM, Aeppli G, Rosenbaum TF (2010). Switchable hardening of a ferromagnet at fixed temperature. Proc. Natl Acad. Sci. USA.

[47] Silevitch DM (2007). A ferromagnet in a continuously tunable random field. Nature.

[48] Tabei SMA, Gingras MJP, Kao Y-J, Stasiak P, Fortin J-Y (2006). Random field effects in the transverse field Ising spin-glass LiHo_x_Y_1−x_F_4_ magnetic material. Phys. Rev. Lett..

[49] Quilliam JA, Meng S, Mugford CGA, Kycia JB (2008). Evidence of spin glass dynamics in dilute LiHo_x_Y_1−x_F_4_. Phys. Rev. Lett..

[50] Tam K-M, Gingras MJP (2009). Spin-glass transition at nonzero temperature in a disordered dipolar Ising system: the case of LiHo_x_Y_1−x_F_4_. Phys. Rev. Lett..

[51] Ghosh S, Rosenbaum TF, Aeppli G, Coppersmith S (2003). Entangled quantum state of magnetic dipoles. Nature.

[52] Bhatt RN, Lee PA (1982). Scaling studies of highly disordered spin-½ antiferromagnetic systems. Phys. Rev. Lett..

[53] Biltmo A, Henelius P (2012). Unreachable glass transition in dilute dipolar magnet. Nat. Commun..

[54] Paalanen MA, Rosenbaum TF, Thomas GA, Bhatt RN (1982). Stress tuning of the metal-insulator transition at Millikelvin temperatures. Phys. Rev. Lett..

[55] Giraud R, Wernsdorfer W, Tkachuk AM, Mailly D, Barbara B (2001). Nuclear spin driven quantum relaxation in LiY_0.998_Ho_0.002_F_4_. Phys. Rev. Lett..

[56] Mennenga G, de Jongh L, Huiskamp W (1984). Field dependent specific heat study of the dipolar Ising ferromagnet LiHoF_4_. J. Magn. Magn. Mater..

[57] Hanggi, P. in *Quantum Transport and Dissipation* 249–286 (Wiley VCH Verlag GmbH, 1998).

[58] Quilliam JA, Meng S, Kycia JB (2012). Experimental phase diagram and dynamics of a dilute dipolar-coupled Ising system. Phys. Rev. B.

[59] Ancona-Torres C, Silevitch DM, Aeppli G, Rosenbaum TF (2008). Quantum and classical glass transitions in LiHo_x_Y_1−x_F_4_. Phys. Rev. Lett..

[60] Ponte P, Papić Z, Huveneers F, Abanin DA (2015). Many-body localization in periodically driven systems. Phys. Rev. Lett..

[61] Fischer MH, Maksymenko M, Altman E (2016). Dynamics of a many-body-localized system coupled to a bath. Phys. Rev. Lett..

