# Supplementary material for: Tuning high-Q nonlinear dynamics in a disordered quantum magnet
Source: Nat Commun. 2019 Sep 5;10:4001. doi: 10.1038/s41467-019-11985-1 (PMC6728381; doi:10.1038/s41467-019-11985-1)
Supplement: Supplementary file 1 — Supplementary Information [file 41467_2019_11985_MOESM1_ESM.pdf]

Supplementary Information for Silevitch et al., “Nonlinear dynamics in a disordered quantum magnet”

Supplementary Note 1: Hole-Burning Background

We start by considering a set of independent clusters, where the effective low-energy Hamiltonian is $H_{\mathrm{le}} = \sum_i H_i$ with

$$H_i = \Delta_i\, \sigma_i^x + M_i\, h(t)\sigma_i^z \quad , \qquad (1)$$

where the sum is over decoupled clusters *i* characterized by an underlying moment M_i and pseudospin operators σ_i , Δ_i corresponds to the splitting between the two states $|\Uparrow\rangle \pm |\Downarrow\rangle$ for cluster *i*, and *h(t)* is an external drive field. If *h(t)* is a time-independent constant *h*>0, the magnetization normalized to M_i is

$$\langle \sigma_i^z \rangle = \frac{1}{2}\frac{M_i h}{\lambda_i} \quad , \qquad (2)$$

where $\lambda_i = \sqrt{\Delta_i^2 + h^2\, M_i^2}$. For $hM_i \ll \Delta_i$, $\langle \sigma_i^z \rangle = \frac{1}{2} M_i h/\Delta_i$ while for $hM_i \gg \Delta_i$, we obtain the expected saturation value of 1/2. Now we consider an oscillating field $h(t) = h\cos(\omega t)$ where for all i, $\hbar\omega \ll \lambda_i$. The full magnetization in this case will then merely be $M(t) = \sum_i M_i < \sigma_i^z > \cos(\omega t)$. If we relax the condition on $\hbar\omega$ but insist that the drive amplitude $h \ll \Delta/M_i$, we obtain the usual linear response form

$$M(t) = \sum_i M_i^2 h \left\{ \left(\frac{\Delta_i}{\Delta_i^2 - (\hbar\omega)^2} \cos(\omega t) \right) + \sin(\omega t)\big(\delta(\hbar\omega - \Delta_i) - \delta(\hbar\omega + \Delta_i)\big) \right\} \qquad (3)$$

where there is now an out-of-phase response which if the frequency is scanned gives the density of states, weighted by M_i^2 for the clusters.

Once we see a continuum in the out-of-phase linear response for a many-body system, a priori we do not know whether we are dealing with a sum of spectra of localized subsystems, as in Eq. (1), for which the eigenfunctions are simply given by direct products of the wavefunctions for the subsystems, or if we are dealing with shorter-lived excitations whose behavior is dominated by coupling between subsystems. A standard method in spectroscopy to determine whether a continuum is due to independent—i.e. localized—two-level systems is to simultaneously relax the conditions on ω and *h* to enter the more interesting and heavily studied regime of driven two-level systems. The idea is to apply a large amplitude field at frequency $\hbar\omega = \Delta_i$ so that

$\langle \sigma_i^z(t) \rangle$ has oscillations of sufficiently large amplitude so that further increments in field can yield only small increases in $\langle \sigma_i^z \rangle$. Without delving into the mathematics of the time-dependent Schrödinger equation[1] for the transverse-field Ising Hamiltonian ((1) in main text), one can convince oneself (a) of the plausibility of this given the magnetization saturation with increasing h in the static limit described by Eq. (2). At the same time, (b) the resonant enhancement of the linear response (3) for ω near $\Delta_i/\hbar$ indicates that we can preferentially excite subsystems *i* with $\omega = \Delta_i$. (a) and (b) together lead to the conclusion that the sample becomes more transparent to radiation at the drive frequency, and a sharp hole is burnt into the spectrum if the two-level system in question cannot interact with two level systems with other values of Δ_i. If such spectral holes can be found in an interacting many-body system, then we have the possibility of probing excited states which are direct products of the excited states for subsystems, meaning that the entire system cannot act as its own heat bath. If there is a weak residual coupling between subsystems, the holes will acquire shapes with width parameters which measure that weak coupling.

Supplementary Note 2: Fano Formalism

The measurements characterize the spectral holes inserted by a nonlinear drive field into the continuum of magnetic excitations in a dense set of interacting dipoles. The large but finite lifetime of the excitations is due to a slight mixing between these (almost perfectly) localized excitations and the continuum formed by their ensemble. The mixing manifests itself in both the decay rate Γ and the Fano *q* parameter (c.f. Eq. (2) in main text). The former is given by Fermi's golden rule:

$$\hbar\Gamma = 2\pi \sum_k V_k^2 \delta(\hbar\omega - E_k) \quad . \tag{4}$$

The q parameter is due to interference between processes taking the ground state of a localized subsystem to an excited state either directly or via another (nearly) localized subsystem[2]:

$$q = \frac{M_{g\alpha} + \mathbb{P}\sum_k \frac{M_{gk}V_k}{(\hbar\omega - E_k)}}{\pi \sum_k M_{gk}V_k \delta(\hbar\omega - E_k)} \quad , \tag{5}$$

where $M_{g\alpha} = \langle g|M|\alpha\rangle$ and $M_{gk} = \langle g|M|k\rangle$ are the matrix elements connecting the ground state to the discrete excited state and to the continuum, respectively. In (4) and (5), V_k is the matrix element connecting the discrete excited state to the kth continuum state, which has energy E_k.[2] Inspection of Eq. (5) allows us to start to understand the zero crossing of q. In the nonlinear regime, the numerator has a modified matrix element $M_{g'\alpha'}$ describing how the longitudinal magnetic field couples the ground and excited states g' and α' as modified by the drive field from g and α , while the denominator contains the product of the analogous matrix element M_{gk} for off-resonant pairs of ground and excited states and the hopping terms V_k between the resonant and off-resonant excited states. It is unlikely that the off-resonant M_{gk} will be much changed by external ac and dc fields, and so—assuming that the principal value term in the numerator cancels to zero—sign changes in q follow from a sign change either in $M_{g'\alpha'}$ or in V_k. A sign change in the latter actually would imply a zero in the former as well because without such a zero, q would diverge at the critical pump (Fig. 5) or transverse (Fig. 6) field h_c. Where all terms in Eq. (5) are analytic near the zero crossing, and with the knowledge that q is linear in h_{pump} and H_t near the zero crossing, we can draw a sharper conclusion, namely that $M_{g\alpha}$ scales like $(h - h_c)^{n+1}$ if V_k scales like $(h - h_c)^n$. To determine n, we can invoke Eq. (4) and the experimental data which show no significant evolution in Γ as a function of the parameters h and H_t, leading to the conclusion that near the zero crossing of q, the exponent characterizing the bath coupling V_k as a function of h and H_t is $n=0$. Therefore, what is driving the zero crossing is only a zero crossing of the matrix element $M_{g\alpha}$ connecting the ground and excited states of the localized subsystems. This means that as h_{pump} crosses h_c, the incremental magnetization δM due to mixing of ground $g(h)$ and excited $\alpha(h)$ states moves from in-phase to out-of-phase with small additional drive fields δh. It is reasonable to believe that where this occurs, the incremental magnetization due to the changing occupancies of the ground and excited states will be highest so that as we observe in the experiment, the zero crossing of q will coincide with the maximum of the total susceptibility which sums diagonal (state occupancy-dominated) and off-diagonal ($M_{g\alpha}$ -dominated) contributions. A further consequence of such considerations is that as the off-diagonal matrix element $M_{g\alpha}$ which accounts for the Fano effect grows from zero, it will also account for an ever larger fraction of the dissipation measured directly at the pump frequency. Considering this mixing allows for proposal of a phenomenological form for the phase angles:

$$\phi'(q) = \tan^{-1}\left(\frac{q^2}{q^2+c^2+\left(h_{\text{pump}}/d\right)^2}\cdot\frac{\chi''_{\text{drive}}(f=f_{\text{pump}})}{\chi'_{\text{drive}}(f=f_{\text{pump}})}\right) \quad (6)$$

Supplemental References

1. Hanggi, P. in *Quantum Transport and Dissipation* 249–286 (Wiley VCH Verlag GmbH, 1998).
2. Riffe, D. M. Classical Fano oscillator. *Phys. Rev. B* **84,** 064308 (2011).
